# Corrosion behavior of self-ligating and conventional metal brackets

**DOI:** 10.1590/2176-9451.19.2.108-114.oar

**Published:** 2014

**Authors:** Lúcio Henrique Esmeraldo Gurgel Maia, Hibernon Lopes Filho, Antônio Carlos de Oliveira Ruellas, Mônica Tirre de Souza Araújo, Delmo Santiago Vaitsman

**Affiliations:** 1 PhD resident of Orthodontics, Federal University of Rio de Janeiro (UFRJ).; 2 Associate professor, Department of Pediatric Dentistry and Orthodontics, UFRJ.; 3 Adjunct professor, Department of Analytical Chemistry, LaDA - IQ/UFRJ.

**Keywords:** Corrosion, Orthodontic brackets, Metal

## Abstract

**Objective:**

To test the null hypothesis that the aging process in self-ligating brackets is not higher than in conventional brackets.

**Methods:**

Twenty-five conventional (GN-3M/Unitek; GE-GAC; VE-Aditek) and 25 self-ligating (SCs-3M/Unitek; INs-GAC; ECs-Aditek) metal brackets from three manufacturers (n = 150) were submitted to aging process in 0.9% NaCl solution at a constant temperature of 37 ± 1ºC for 21 days. The content of nickel, chromium and iron ions in the solution collected at intervals of 7, 14 and 21 days was quantified by atomic absorption spectrophotometry. After the aging process, the brackets were analyzed by scanning electron microscopy (SEM) under 22X and 1,000X magnifications.

**Results:**

Comparison of metal release in self-ligating and conventional brackets from the same manufacturer proved that the SCs group released more nickel (p < 0.05) than the GN group after 7 and 14 days, but less chromium (p < 0.05) after 14 days and less iron (p < 0.05) at the three experimental time intervals. The INs group released less iron (p < 0.05) than the GE group after 7 days and less nickel, chromium and iron (p < 0.05) after 14 and 21 days. The ECs group released more nickel, chromium and iron (p < 0.05) than the VE group after 14 days, but released less nickel and chromium (p < 0.05) after 7 days and less chromium and iron (p < 0.05) after 21 days. The SEM analysis revealed alterations on surface topography of conventional and self-ligating brackets.

**Conclusions:**

The aging process in self-ligating brackets was not greater than in conventional brackets from the same manufacturer. The null hypothesis was accepted.

## Abstract (pt)

**Objetivo:**

testar a hipótese nula de que o processo de envelhecimento em braquetes autoligáveis não é superior ao de braquetes convencionais.

**Métodos:**

Vinte e cinco braquetes metálicos convencionais (GN, 3M/Unitek; GE, GAC; VE, Aditek) e 25 autoligáveis (SCs, 3M/Unitek; INs, GAC; ECs, Aditek) de três fabricantes (n = 150) foram submetidos ao envelhecimento em solução de NaCl à temperatura constante de 37 ± 1ºC, durante 21 dias. O conteúdo de íons níquel, cromo e ferro na solução coletada com 7, 14 e 21 dias foi quantificado por meio de espectrofotometria de absorção atômica. Depois de completado o processo de envelhecimento, os braquetes foram analisados com microscópio eletrônico de varredura (MEV), em magnificações de 22x e de 1.000x.

**Resultados:**

comparando-se a liberação de metais por braquetes autoligáveis e convencionais do mesmo fabricante, observou-se que o grupo SCs liberou mais níquel (p < 0,05) que o grupo GN após 7 e 14 dias, mas menos cromo (p < 0,05) após 14 dias e menos ferro (p < 0,05) nos três períodos experimentais. O grupo INs liberou menos ferro (p < 0,05) que o grupo GE após 7 dias, e menos níquel, cromo e ferro (p < 0,05) após 14 e 21 dias. O grupo ECs liberou mais níquel, cromo e ferro (p < 0,05) que o grupo VE após 14 dias; entretanto, liberou menos níquel e cromo (p < 0,05) após 7 dias e menos cromo e ferro (p < 0,05) após 21 dias. A análise no MEV demonstrou alterações na topografia da superfície de braquetes convencionais e autoligáveis.

**Conclusões:**

o processo de envelhecimento em braquetes autoligáveis não foi superior ao de braquetes convencionais do mesmo fabricante. A hipótese nula está aceita.

## INTRODUCTION

Metal alloys are frequently used in Orthodontics to fabricate brackets, bands, wires and tubes. These alloys are made of austenitic stainless steel,^1,2^ such as AISI 303, 304 and 316L,^3^ and have nickel, chromium and iron in their composition.

When exposed to the oral environment, metal orthodontic accessories are subjected to degradation, such as corrosion by pits, fracture due to fatigue, increase in the coefficient of friction or microbiological degradation.^4,5^ When the corrosive process occurs, metal ions are released into the oral medium or transformed into oxides.^2^

Self-ligating brackets have been widely used during the last decade by supposing that they have advantages^6^ such as reduced treatment time as a result of reduced friction, and a more irregular morphology due to the connection system and larger volume in comparison with conventional brackets. For this reason, it is possible that they are more susceptible to corrosion.

Orthodontic appliance biodegradation is undesirable, and can hinder sliding mechanics,^7^ cause reactions of hypersensitivity due to the release of nickel and chromium,^8,9^ stain the enamel as a result of incorporating metal ions,^10^ or even damage the appliance.^2^ In self-ligating brackets, corrosion is also capable of altering the connection system and reducing its effectiveness.^11^ In the active ligation system, it may hinder the capacity of pressing the wire into the slot.^11^ Conversely, in the passive one, it may hamper opening or closing of the connection system.^12^

The aim of this study was to assess the aging process in conventional and self-ligating metal brackets. To this end, the null hypothesis assumed that the aging process in self-ligating brackets is not greater than in conventional brackets.

## MATERIAL AND METHODS

Table 1 shows the distribution of the analyzed samples. The sample consisted of 150 metal brackets, 75 self-ligating and 75 conventional for maxillary right central incisors, from three different manufacturers (3M Unitek, Aditek and GAC) (25 brackets each). The conventional brackets were Gemini (3M/Unitek, Monrovia, CA, USA), Generus (GAC, Islandia, NY, USA) and Vector (Aditek, Cravinhos, SP, Brazil); whereas the self-ligating brackets were Smart Clip (3M/Unitek), In Ovation R (GAC) and Easy Clip (Aditek).

**Table 1:** Distribution of samples.

Bracket	n	Group	Ligation system	Manufacturer	Reference	Lot
Gemini	25	GN	Conventional	3M / Unitek	119-713	019329500
Generus	25	GE		GAC	31-212-32	B479
Vector	25	VE		Aditek	12.32.411	100329
Smart clip	25	SCs	Self ligating	3M / Unitek	004-302	016560600B
In Ovation R	25	INs		GAC	89-112-00	B3Y7
Easy clip	25	ECs		Aditek	13.32.011	090303

The 25 brackets of each group were divided into five subgroups, with an equal number of samples each, numbered from one to five.

The brackets of each subgroup were stored in previously sterilized Petri dishes, without coming into contact with one another. They were subjected to corrosion in 20 ml of sterile 0.9% NaCl solution^13,14^ for a period of 21 days. The Petri dishes were kept in an incubator (Quimis - Quimis Aparelhos Científicos LTDA., Diadema-SP, Brazil) at an unchanged temperature of 37 ± 1ºC.^15^ Every 7 days ± 1 hour, the brackets were removed and transferred to another container filled with a new solution.^1,13^

At the end of each experimental time interval (7 days, 14 days and 21 days), the solution remaining in each container was analyzed by atomic absorption spectrophotometry in a Varian spectrophotometer, Model AA-1475 (Varian Indústria e Comércio Ltda., São Paulo/SP, Brazil) with a view to determining their nickel, chromium and iron contents.

At the end of the experiment, five brackets from each group (one from each subgroup) were randomly selected and their surface topography was analyzed by scanning electron microscopy - SEM (Jeol JSM 6460 LV, Japan) and compared with the surface of new brackets.

SEM was operated at 20 kV, and readouts were taken under 22X and 1,000X magnification. The 22X magnification allowed a complete view of the bracket. The 1,000X magnification was performed at the slot of each bracket with the connection system opened so as to analyze the area for wire insertion. No treatment was performed on the brackets at the end of the experiment in order to prevent any possible oxides, deposited on the bracket surface as a result of the corrosion process, from being removed. Energy-dispersive X-ray spectroscopy (EDS) was used to identify atypical depositions on bracket surface.

In each group and at each time interval, nickel, chromium and iron concentrations as well as the total amount released during the experiment were statistically assessed. The Kolmogorov-Smirnov test was used to verify the sample normality of distribution. As normal distribution was not found, the non-parametric ANOVA test with Tukey post-test were applied for intragroup assessment, whereas the Kruskal-Wallis test with Wilcoxon post-test were applied for intergroup assessment. The data were statistically analyzed using SPSS 17.0 software (Statistical Package for Social Sciences, SPSS Inc., Chicago, IL, USA). The significance level was set at 5%.

## RESULTS

During the experimental period, the release of nickel, chromium and iron metal ions was observed in all groups, except for nickel ion in the GN group, which was not detected at any of the time intervals (Tables 2, 3 and 4).

**Table 2:** Nickel content (ppm) after the different experimental time intervals.

Nickel release (PPM)											
	7 days			14 days			21 days			Total	
Group	Mean ± SD	Intra. Sig.	Inter. Sig.	Mean ± SD	Intra. Sig.	Inter. Sig.	Mean ± SD	Intra. Sig.	Inter. Sig.	Mean ± SD	Inter. Sig.
GN	0.00	^a^	^A^	0.00	^a^	^A^	0.00	^a^	^A^	0.0 (0.0)	^A^
GE	0.14 ± 0.28	^a^	^ABC^	0.92 ± 0.03	^b^	^B^	0.68 ± 0.02	^b^	^B^	1.74 ± 0.27	^B^
VE	0.53 ± 0.03	^a^	^B^	0.00 ± 0.01	^b^	^A^	0.00 ± 0.01	^b^	^A^	0.54 ± 0.03	^C^
SCs	0.11 ± 0.03	^a^	^C^	0.07 ± 0.01	^b^	^C^	0.00	^c^	^A^	0.18 ± 0.01	^D^
INs	0.14 ± 0.01	^a^	^C^	0.39 ± 0.02	^b^	^D^	0.00 ± 0.01	^c^	^A^	0.54 ± 0.03	^C^
ECs	2.79 ± 0.03	^a^	^D^	1.24 ± 0.03	^b^	^E^	0.00 ± 0.01	^c^	^A^	4.03 ± 0.03	^E^

Identical letters reveal no statistical difference (p > 0.05). Intragroup significance - Comparison of the different time intervals in each group with ANOVA test and Tukey post hoc-test. Intergroup Significance - Comparison among the groups in each time interval with Kruskal-Wallis test and Wilcoxon post hoc-test.

**Table 3:** Chromium content (ppm) after the different experimental time intervals.

Chromium release(PPM)											
	7 days			14 days			21 days		Total		
Group	Mean ± SD	Intra. Sig.	Inter. Sig.	Mean ± SD	Intra. Sig.	Inter. Sig.	Mean ± SD	Intra. Sig.	Inter. Sig.	Mean ± SD	Inter. Sig.
GN	0.50 ± 0.11	^a^	^AB^	1.03 ± 0.01	^b^	^A^	1.68 ± 0.03	^c^	^A^	3.21 ± 0.12	^AB^
GE	0.89 ± 0.10	^a^	^C^	1.29 ± 0.02	^b^	^B^	1.95 ± 0.03	^c^	^B^	4.13 ± 0.10	^C^
VE	1.15 ± 0.02	^a^	^D^	1.03 ± 0.05	^b^	^A^	1.95 ± 0.06	^c^	^B^	4.13 ± 0.11	^C^
SCs	0.45 ± 0.01	^a^	^A^	0.30 ± 0.03	^a^	^C^	2.05 ± 0.35	^b^	^AB^	2.81 ± 0.33	^D^
INs	0.76 ± 0.02	^a^	^C^	1.03 ± 0.08	^b^	^A^	1.42 ± 0.02	^c^	^C^	3.21 ± 0.09	^A^
ECs	0.63 ± 0.02	^a^	^B^	1.55 ± 0.03	^b^	^D^	1.16 ± 0.03	^c^	^D^	3.34 ± 0.05	^B^

Identical letters indicate no statistical difference (p > 0.05). Intragroup significance - Comparison of the different time intervals in each group with ANOVA test and Tukey post hoc-test. Intergroup Significance - Comparison among the groups in each time interval with Kruskal-Wallis test and Wilcoxon post hoc-test

**Table 4:** Iron content (ppm) after the different experimental time intervals.

Iron release (ppm)											
	7 days			14 days			21 days		Total		
Group	Mean ± SD	Intra. Sig.	Inter. Sig.	Mean ± SD	Intra. Sig.	Inter. Sig.	Mean ± SD	Intra. Sig.	Inter. Sig.	Mean ± SD	Inter. Sig.
GN	0.35 ± 0.03	^a^	^A^	0.44 ± 0.03	^b^	^A^	1.02 ± 0.02	^c^	^A^	1.81 ± 0.04	^A^
GE	0.93 ± 0.05	^a^	^B^	1.02 ± 0.02	^b^	^B^	1.28 ± 0.02	^c^	^B^	3.23 ± 0.08	^B^
VE	1.01± 0.05	^a^	^B^	0.77 ± 0.04	^b^	^C^	1.02 ± 0.02	^a^	^A^	2.80 ± 0.09	^CD^
SCs	0.00 ± 0.01	^a^	^C^	0.01 ± 0.02	^a^	^D^	0.94 ± 0.02	^b^	^C^	0.96 ± 0.04	^E^
INs	0.77 ± 0.02	^a^	^D^	0.94 ± 0.02	^b^	^E^	1.01 ± 0.03	^c^	^A^	2.72 ± 0.02	^C^
ECs	1.02 ± 0.03	^a^	^B^	0.94 ± 0.02	^b^	^E^	0.94 ± 0.04	^b^	^D^	2.90 ± 0.08	^D^

Identical letters indicate no statistical difference (p > 0.05). Intragroup significance - Comparison of the different time intervals in each group with ANOVA test and Tukey post hoc-test. Intergroup Significance - Comparison among the groups in each time interval with Kruskal-Wallis test and Wilcoxon post hoc-test.

There was greater release of nickel in the initial period of the experiment, both for conventional and self-ligating brackets, with a trend towards no release of this ion at the end of the experiment (p < 0.05). Only group GN and group GE behaved differently with greater release of nickel on the 14^th^ and 21^st^ days. The experimental groups revealed a trend towards increasing release of ions, such as chromium and iron, from the first to the third week of the experiment (p < 0.05). The only exception was group VE, in which a great amount of iron release was found in the first week. Metal release was similar in self-ligating and conventional brackets from the same manufacturer. Group SCs released more nickel (p < 0.05) than group GN after 7 and 14 days, but group GN released more chromium (p < 0.05) after 14 days and more iron (p < 0.05) at the three experimental time intervals. Group INs released less iron (p < 0.05) than group GE after 7 days; and less nickel, chromium and iron (p < 0.05) after 14 and 21 days. Group ECs released more nickel, chromium and iron (p < 0.05) than group VE after 14 days, but released less nickel and chromium (p < 0.05) after 7 days and less chromium and iron (p < 0.05) after 21 days.

**Figure 1:**
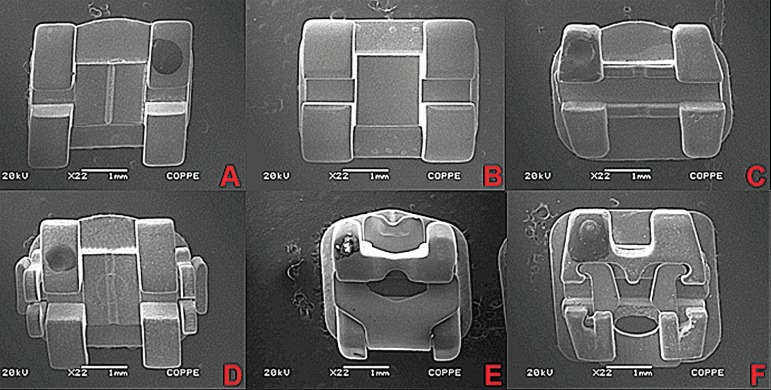
Surface topography of new brackets visualized by SEM under 22X magnification. **A**) GN; **B**) GE; **C**) VE; **D**) SCs; **E**) INs; **F**) ECs.

**Figure 2:**
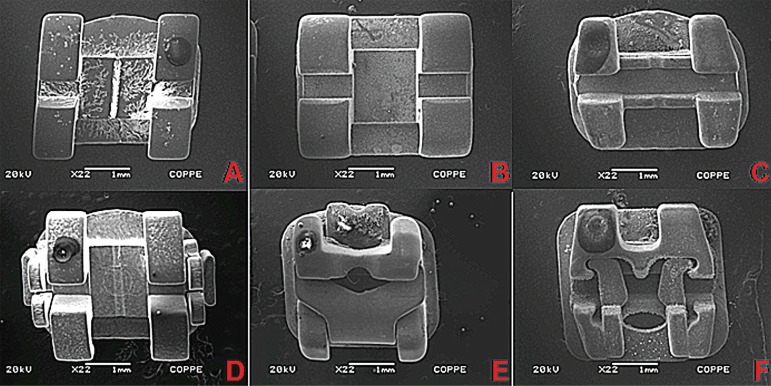
Surface topography of brackets visualized by SEM under 22X magnification after 21 days. **A**) GN; **B**) GE; **C**) VE; **D**) SCs; **E**) INs; **F**) ECs.

**Figure 3:**
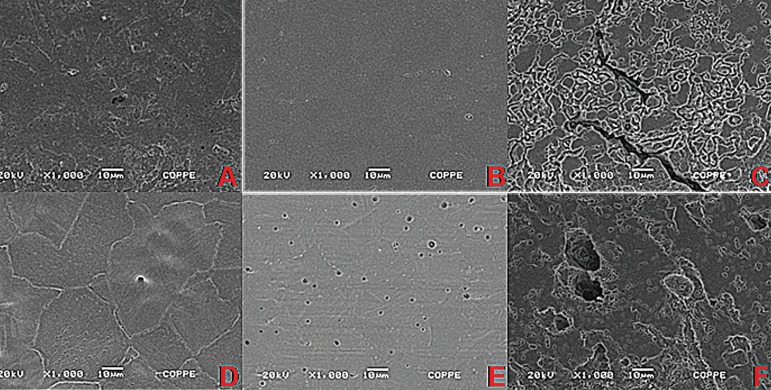
Surface topography of new brackets visualized by SEM under 1000X magnification. **A**) GN; **B**) GE; **C**) VE; **D**) SCs; **E**) INs; **F**) ECs.

**Figure 4:**
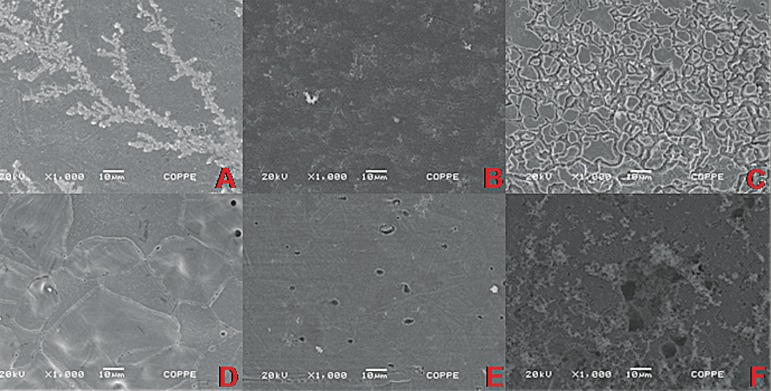
Surface topography of brackets visualized by SEM under 1000X magnification after 21 days. **A**) GN; **B**) GE; **C**) VE; **D**) SCs; **E**) INs; **F**) ECs.

At the end of the experiment, the SEM analysis revealed that the surface topography of all brackets changed, with signs of aging when compared with the surface of new brackets (Figs 1 to 4).

## DISCUSSION

Bracket corrosion with consequent release of metal ions during orthodontic treatment may hinder orthodontic mechanics,^2^ trigger hypersensitivity reactions with hyperplasia and gingival tissue inflammation,^4,8,9^ and contribute to iatrogenic staining of the enamel with the incorporation of metals.^10,16^

A corroded bracket often presents a more irregular surface and can accumulate products resulting from corrosion (Fig 4). Thus, friction between the bracket and wire during sliding mechanics increases, making it necessary to apply force of greater magnitude to overcome the friction and produce physiologic tooth movement.^7,17,18^ When excessive force is applied to the bracket, there is loss of mechanical control and increased probability of root resorption.^5,9,19,21^

One of the advantages attributed to self-ligating brackets is the lower degree of friction that the ligation system imposes on the orthodontic wire when compared with conventional tying.^6,22,24^ However, if the bracket has an increased corrosion potential, friction also increases,^25^ affecting this advantage.

In the present study, SEM analysis under 22X magnification (Fig 2) revealed that, in general, self-ligating brackets showed more areas with altered surface topography due to corrosion, when compared with conventional brackets from the same manufacturer, possibly due to their geometry with larger retentive areas. Thus, brackets in the INs group had a more irregular surface than the brackets in the GE group, while the brackets in the ECs group presented greater alterations in comparison to those in the VE group. The self-ligating brackets of the SCs group and the conventional brackets in group GN did not present significant alterations in their surface topography. Energy-dispersive X-ray spectroscopy (EDS) revealed NaCl deposition on the surfaces of the brackets in group GN.

Analysis of the images obtained by SEM under 1000X magnification (Figs 3 and 4) suggests that new brackets with more irregular surfaces were more susceptible to corrosion. Thus, the brackets in group ECs, which presented a surface topography with deficient smoothness and porosity at the beginning of the experiment, presented a considerably more irregular surface at the end of it. The brackets in group VE also presented considerably altered surfaces at the end of the experiment, due to the formation of oxides. Moreover, the brackets in group INs, which presented irregularities at the beginning of the experiment, were significantly subjected to pitting corrosion. Quantification of the metal ions released during the experiment corroborates these data. These groups had great release of nickel, chromium and iron ions.

The release of metal ions into the oral environment may trigger hypersensitivity reactions.^4,5,8,9,26^ Nickel and chromium are present in the composition of brackets with the goal of increasing resistance to corrosion.^2,4,16,27^ These elements are largely responsible for the aforementioned adverse reactions. Nickel is strongly responsible for triggering more allergic reactions than any other metal.^8,26^

Under the conditions of this experiment, the release of nickel was considerably lower than the daily ingestion of this metal through food (300 to 600 µg/day).^13^ However, it is worth noting that susceptible patients in contact with small concentrations of this metal are more likely to suffer hypersensitivity reactions.^29^

The oral reaction of allergy to nickel is difficult to diagnose, since its clinical signs and symptoms are similar to those of gingivitis caused by poor oral hygiene. The low number of reports of hypersensitivity to nickel is possibly due to this difficulty in diagnosis. Epidemiological data point towards an incidence of sensitivity to this metal of approximately 20% in the general population.^28,30^

Similarly to other *in vitro* studies,^1,15,31^ the release of nickel in this experiment was higher in the first two weeks, with a trend towards no further release during the third week. The exception was group GE which continued to release nickel ions during the last week (Table 2).

Similarly to previous studies,^1,13^ chromium and iron ions had a trend towards increasing release throughout the experiment in all groups, except for group ECs (Tables 3 and 4). In this group, there was a greater release of chromium in the second week, and greater release of iron in the first week, although the concentration of these ions remained high at the end of the third week.

Within the limitations and conditions of this experiment, it is reasonable to conclude that metal release was similar in self-ligating and conventional brackets from the same manufacturer.

When self-ligating and conventional brackets from the same manufacturer were compared, group SCs proved to release more nickel (p < 0.05) than group GN after 7 and 14 days, whereas group GN released more chromium (p < 0.05) after 14 days and more iron (p < 0.05) at the three experimental time intervals. Group INs released less iron (p < 0.05) than group GE after 7 days and less nickel, chromium and iron (p < 0.05) after 14 and 21 days. Group ECs released more nickel, chromium and iron (p < 0.05) than group VE after 14 days, but released less nickel and chromium (p < 0.05) after 7 days and less chromium and iron (p < 0.05) after 21 days.

Metal ions released by metal brackets and bands during orthodontic treatment may be incorporated into tooth enamel, causing iatrogenic color alteration and stains.^10,16^ In cases of severe pigmentation, restorative treatment of the vestibular surface of the stained tooth proves necessary.^32,33^ Special care must be given to patients with poor oral hygiene, since altered oral environment, with reduced pH and presence of acidogenic microorganisms potentiates the corrosion of metal accessories. Additionally, enamel demineralization and remineralization processes may influence the incorporation of metals.^4,10^

## CONCLUSIONS

» The null hypothesis was accepted.
» The SEM analysis revealed that self-ligating and conventional brackets presented signs of aging.
» Metal ions release in self-ligating brackets was similar to conventional brackets from the same manufacturer.
